# Effect of Flapless Immediate Implantation and Filling the Buccal Gap with Xenograft Material on the Buccal Bone Level: A Randomized Clinical Trial

**Published:** 2017-11

**Authors:** Mojgan Paknejad, Solmaz Akbari, Hoori Aslroosta, Mehrdad Panjnoush, Samira Hajheidary

**Affiliations:** 1 Professor, Dental Research Center, Dentistry Research Institute, Tehran University of Medical Sciences, Tehran, Iran; Department of Periodontics, School of Dentistry, Tehran University of Medical Sciences, Tehran, Iran; 2 Assistant Professor, Department of Periodontics, School of Dentistry, Tehran University of Medical Sciences, Tehran, Iran; 3 Associate Professor, Department of Oral and Maxillofacial Radiology, School of Dentistry, Tehran University of Medical Sciences, Tehran, Iran; 4 Postgraduate Student, Department of Periodontics, School of Dentistry, Tehran University of Medical Sciences, Tehran, Iran

**Keywords:** Dental Implants, Maxilla, Tooth Socket, Heterografts

## Abstract

**Objectives:**

Following tooth extraction, soft and hard tissue alterations occur; Different factors can affect this process. The objective of this study was to determine the effect of gap filling on buccal alveolar crestal bone level after immediate implant placement after 4- to 6-month observation period.

**Materials and Methods:**

This randomized clinical trial was performed on 20 patients (mean age of 38.8 years) requiring tooth extraction in a total of 27 areas in the anterior maxilla. The treatment strategy was as follows: atraumatic flapless tooth extraction, implant placement, insertion of a graft (test group) or no material (control group) between the implant and the socket wall, connection healing abutment placement and suturing the area. Clinical and cone beam computed tomographic examinations were performed before implant placement (baseline), 24 hours after surgery and 4-6 months (T2) after implant placement, to assess the buccal plate height (BH) and implant complications.

**Results:**

After 4 months of healing, a reduction in different bone measurements was noticed in the two groups. No statistically significant differences were assessed in bone height measurements between the test and control groups at different time points. The study demonstrated that immediate implantation resulted in 1.30 and 1.66 mm reduction in buccal bone plate in the test and control groups, respectively.

**Conclusions:**

The study demonstrated that immediate implantation in the extraction socket together with xenograft failed to prevent bone resorption.

## INTRODUCTION

Immediate implant placement in the anterior maxilla is documented as a highly predictable, successful procedure. However, it is challenging due to the demand for well-anchored implants and satisfactory aesthetic results [1,2]. This protocol was first presented in 1976 by Schulte and Heimke [3]. Several advantages have been mentioned for this procedure, such as the reduced overall treatment time, the reduced number of surgical procedures and the optimal availability of the existing bone for implant insertion [4,5]. The physiological healing process and remodeling of the alveolar bone is accompanied by changes in the surrounding soft and hard tissues which can be more challenging in the anterior maxilla due to the demand for satisfactory aesthetic results [6–8]. Based on several studies, it is well accepted that implant placement into a fresh extraction socket does not counteract physiological bone remodeling in the alveolar bone crest. Therefore, both vertical and horizontal dimensional changes of the alveolar crest may be expected [9]. Several factors affect the resorption of alveolar bone crest in immediate implantation including the thickness of the buccal bone wall, the gingival thickness, flap or flapless technique, distance from the implant platform to the crestal bone, surface coating and designs and the size of gap between the implant and the wall of the alveolar socket [9–11]. After implant insertion, the distance between the walls and surface of implant may require augmentation to predictably achieve bone-implant contact and prevent soft tissue ingrowth associated with natural socket healing [12].

The effect of different materials in regenerative therapies combined with immediate implantation has been evaluated in several clinical studies. Araujo et al, [13,14] in animal studies demonstrated that socket grafting with deproteinized bovine bone mineral preserved the dimensions of the alveolar ridge.

This finding was in accordance with Nevins et al, [15] in 2006 who studied on immediate implantation in the anterior maxilla and filled the gap with deproteinized bovine bone mineral and demonstrated less resorption of the buccal plate compared to the non-grafted control sites. In a systematic review by Vignoletti et al, [16] in 2012 on ridge preservation after tooth extraction, it was concluded that less vertical and horizontal contraction of the bone crest may be the result of socket grafting with biomaterial. Although many authors have advocated the use of bone materials with immediate implant placement, evidence supporting this additional treatment from controlled clinical trials is lacking [17–19].

The hypothesis was that the usage of xenograft in the gap could have ideal results. The objective of this study was to determine the effect of xenograft bone material on the buccal bone level changes of the alveolar ridge. We specifically measured the distance from the buccal bone crest to the implant platform immediately after placement and 4 months later on cone beam computed tomography (CBCT) images.

## MATERIALS AND METHODS

### Recruitment

This randomized clinical trial was conducted in accordance with the guidelines laid down in the Declaration of Helsinki (1964), and all procedures involving humans were approved by the ethics committee of Tehran University of Medical Sciences. Written informed consent was obtained from all subjects prior to the start of the trial and the study was registered in the Iranian Registry of Clinical Trials (available at: http://www.irct.ir, identifier: IRCT ID: IRCT201606188898N3).

The study population consisted of all patients who presented to the Tehran University of Medical Sciences between 2015–2016 requiring extraction of upper incisors, lateral incisors or premolars (teeth #15-25) in whom immediate placement of implant was possible. Indications for tooth extraction were factors that could cause hopeless prognosis, such as un-restorable carious lesions, endodontic treatment failure and root or crown fractures. For inclusion in the study, patients had to meet the following criteria: age between 18 to 50 years, good oral hygiene defined as full mouth plaque score 25% [7], requiring extraction of teeth #15–25 in the premolar region and the anterior maxilla, a keratinized gingival band 3 to 5 mm wide, presence of intact buccal bone plate, and adequate bone height apical to the alveolus of the failing tooth to ensure primary implant stability of at least 35 Ncm. The exclusion criteria were: Patients with uncompensated systemic diseases or any systemic disease that could interfere with implant treatment, smokers, active infections in the surgical site, or presence of acute periapical lesions, patients with periodontal diseases, probing depth >4 mm at the adjacent teeth and poor oral hygiene.

### Surgical procedure:

Before surgery, comprehensive clinical and radiographic examination was performed by two experienced clinicians. The initial CBCT scan of the patients helped us confirm the presence of buccal bone. One week before the surgery, we made alginate impressions from all patients, who met the inclusion criteria before surgery and then a radiographic stent with metallic wire at the center of the tooth in mesio-distal direction was made on patients’ casts using auto-polymerizing acrylic resin. Therefore, we could evaluate the same region on CBCT scans at different evaluation time points. Prior to surgery, patients rinsed 2 cc of 0.2% chlorhexidine gluconate mouthwash (Behsa, Tehran, Iran). The immediate implant placement protocol has been thoroughly described in the dental literature [20,21] and the protocol was performed according to the criteria set by Buser et al, [22]. The surgical procedure was performed under local anesthesia. The extraction was carried out with the use of a periotome (Hu-Friedy, Chicago, USA). and forceps as atraumatically as possible to avoid damaging the bony socket without elevation of mucoperiosteal flap and followed by thorough degranulation of any soft tissue remnants and ensuring the integrity of the buccal bone plate. Standard drilling procedures were performed according to the manufacturer’s instructions so that lateral contact could be achieved with the implant body.

The platforms of the implants were located 1.0–2.0 mm below the buccal bone crestal level and then the gap between the buccal bone and the surface of the implants was filled by xenograft material or left empty. We divided the patients into four groups as premolar or non-premolar cases and the width of the gap between the fixture and the inner surface of the socket as gap ≥2mm or <2mm. Random allocation of xenograft to the site was done by drawing envelopes. Stratified balanced block randomization was used in order for appropriate distribution of tooth location in the two groups.

After implant placement and obtaining primary stability, a randomization envelope was opened and the xenograft (CompactBone® B, Dentegris GmbH, Duisburg, Germany) was randomly placed in the area.

Next, the peri-implant mucosa was sutured. Implants 12–14 mm in length and 3.5–4.3 mm in diameter were used according to the requirement. The healing abutments (Dentium Implants, Implantium, Seoul, Korea) were connected. Patients were instructed to rinse chlorhexidine twice daily during the healing phase, 500 mg amoxicillin every 8 hours for 7 days and novafen every 6 hours for pain relief.

Two weeks after implant placement, the patients returned to the clinic for post-operative control. After 4 months, the final crown was placed on the abutment and the patients were referred to the Radiology Department of Tehran University of Medical Sciences for CBCT.

### Analysis of the CBCT sections:

All CBCT scans were taken in the Radiology Department of Tehran University of Medical Sciences. The scans were obtained using Alphard VEGA scanner (Model: Alphard-3030, Asahi Roentgen Inc. Co., Kyoto, Japan) with 0.2 mm voxel size. Parameters were set at 4.0 mA and 110 kV with 102×102 mm field of view. The exposure time was 17 seconds and images were acquired using Planmeca Romexis TM Imaging Software 2.2. These values were the same (standard) for all samples. The changes of buccal bone height were assessed on CBCT scans at three time points: pre-operatively (T0), 24 hours after surgery (T1), and 4–6 months postoperatively (T2). Linear measurements of the bone height from the crestal point of the buccal bone plate to the platform of the implants were made by 2 radiologists on cross sectional views in the middle of the maxillary central and lateral incisors, canine and premolar sites using Romexis Planmeca Viewer software version 2.2. [23,24].

For this purpose, we placed the implant in a straight line in panoramic view; thus, the cut lines were perpendicular to the sections along the fixture in the image. If the cut is skewed, accurate measurement cannot be made.

**Fig. 1:**
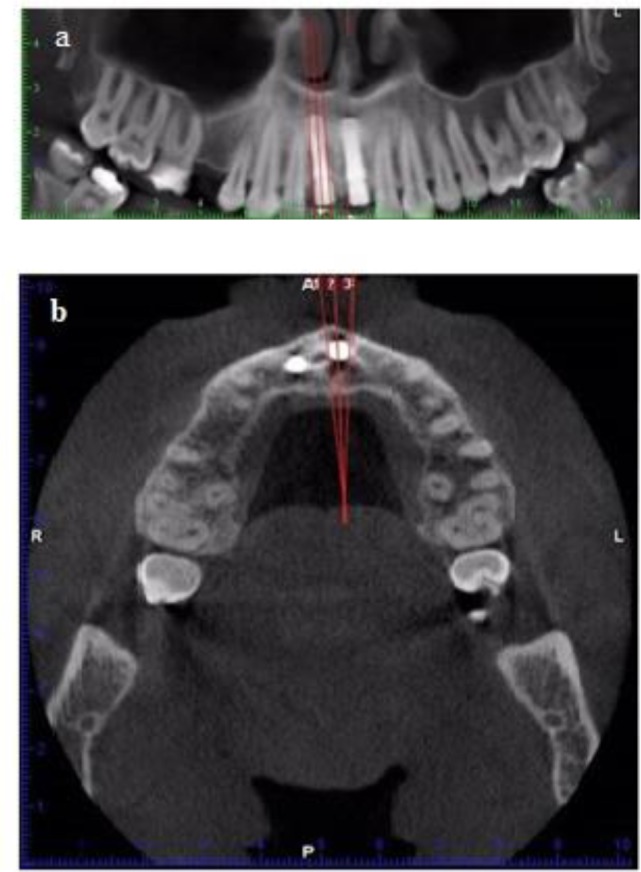
(a) The parallel lines in the mesial, central and distal of each implant were drawn on panoramic and (b) axial sections

**Fig. 2:**
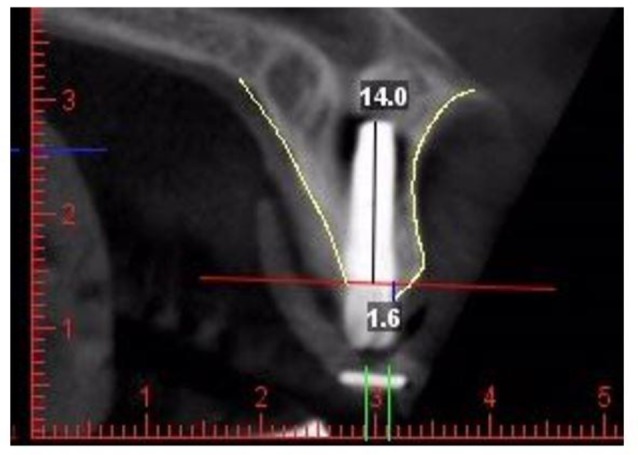
Measurements of bone height from crestal point of the buccal bone plate to the platform of the implant on the midbuccal cross-sectional cone beam computed tomography slice after a straight line was drawn in the direction of the long axis of implant and another in the platform region

Based on the width of implants, cut lines with 1 to 2 mm widths in the central, distal and mesial regions of the implants were drawn (Fig. 1); thus, the central cut in the area of metal wire was selected. In order to accurately see the bone, contrast and brightness of images were adjusted. The images were processed two-dimensionally in Planmeca Romexis TM Imaging Software 2.2. First, one line was drawn along the body of the implant and then the second line was drawn perpendicular to the first line along the platform, and the most coronal point on the crest was determined. Therefore, the distance between the platform and the most coronal point on the bone crest was measured (Fig. 2).

### Statistical analysis:

The data were analyzed using SPSS version 21 software (SPSS Inc., IL, USA). Normal distribution of quantitative data was assessed graphically using one-sample Kolmogorov- Smirnov test.

To compare the changes in size of the two groups, independent t-test was used. Paired t-test was used for the comparison of the mean of all variables in order to identify within- group differences. P<0.05 was considered significant.

## RESULTS

Of a total of 20 patients who underwent immediate flapless implant placement, three males and 17 females with a mean age of 38.8 years (range 37–57 years) were followed up for a median period of 4–6 mouths. Five patients were excluded during surgery because the socket wall was not intact after exploring it by a probe after tooth extraction; although the buccal bone had shown good integrity on CBCT scans. A total of 27 implants (14 test and 13 control implants) were inserted and their distribution is shown in Table 1. Throughout the study, all surgical sites healed uneventfully with no signs of gingival inflammation or infection. One patient had severe pain during the first week after surgery but the pain subsided after two weeks and the implant integrated without any further intervention.

**Table 1.** Frequency distribution of the implant site

	**Incisors N(%)**	**Canine N(%)**	**Premolars N(%)**
**Test (n=14)**	3 (21)	4 (28)	7 (50)
**Control (n=13)**	4 (30)	1 (7)	8 (61)

### Changes in level of bone height in the two groups:

**Fig. 3:**
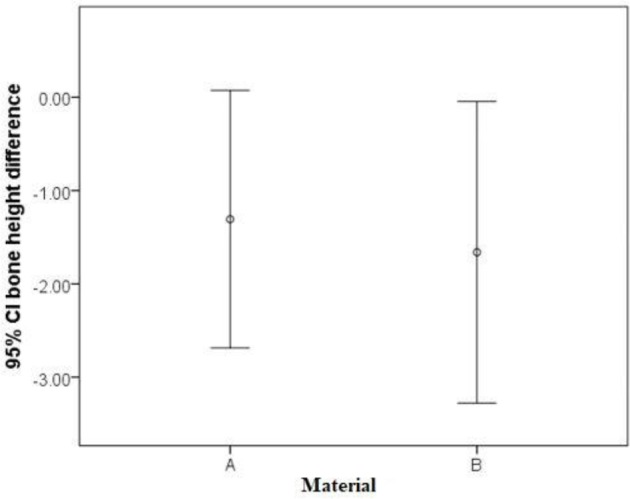
Differences between the two groups with (A)or without (B)material after 4–6 months of follow up

Table 2 shows the changes in bone height in the two study groups. At baseline, the mean distance between the most coronal point on the buccal alveolar bone crest and the implant platform was 1.92 mm and 0.95 mm in the test and control groups, respectively. After 4–6 months of follow-up, the mean reduction in buccal alveolar bone height was −1.30 mm in the test and −1.66 mm in the control group. No significant difference was observed in bone height reduction between the two groups (independent t-test, P= 0.71, Fig. 3).

**Table 2.** Measurements of the bone height at baseline and after four months in the two groups

	**Test group (n=14)**	**Control group (n=13)**	**P-value**
**Bone height baseline**	1.92±1.08	0.95±0.96	0.02
**Bone height after 4–6 months**	0.61±2.61	−0.71±2.86	0.22
**Bone height differences**	−1.30±2.38	−1.66±2.67	0.72
**P-value^a^**	0.06^a^	0.04^a^	

## DISCUSSION

In the present study, vertical reduction in the buccal bone plate following flapless immediate implant placement in the anterior maxilla was evaluated on CBCT scans and the effect of buccal gap filling with slow resorption bone material on the reduction of buccal bone height was evaluated [25–27]. Our results showed that after 4–6 months of healing, the height of buccal bone wall undergoes a mean reduction of 1.30 and 1.66 mm in the test and control groups, respectively. Botticelli et al, [28] reported that after immediate implantation in the anterior maxilla, approximately 56% of the buccal and 30% of the lingual bone walls of the sockets underwent horizontal resorption. The highest dimensional changes of the ridge occurred during the first year especially within the first three months after tooth extraction [29,30]. However, the factors that can affect the dimensional changes of the facial bone and soft tissue breakdown have not been clearly identified [24]. Several studies investigated the dimension of the buccal bone in grafted [28,31] and non-grafted [6,32,33] types of immediate implantation and reported a mean reduction of the buccal bone height to be 0.5–1 mm at 4–6 months after surgery. Degidi et al, [34] also showed the mean vertical crest reduction of about 0.76 ± 0.96 mm. deM Sartori et al. [35] studied immediate implantation in the anterior maxilla subjected to immediate loading without using any bone material. They reported a mean resorption of 3.31 mm in the apical direction in the buccal bone crest after 6 months. Sanz et al. [36] demonstrated that the mean vertical crest reduction in immediate implantation without using graft material was about 1mm at the buccal and 0.5 mm at the palatal aspects. A systematic review by Clementini et al. [37] showed interesting results and revealed that after immediate implant insertion alone, the buccal bone plate underwent horizontal and vertical reduction of about 1mm. These results were in accordance with another systematic review by Lee et al. [38] analyzing bone dimensional changes after immediate implant placement and reporting approximately 0.5–1.0 mm reduction in vertical and horizontal aspects after 4–12 months following surgery. Recently, Benic et al. [25] studied the dimensional changes of the facial bone by CBCT after socket grafting with xenogeneic bone substitute and covering with collagen membrane. They found no correlation between bone defects at baseline and vertical dimensions of the facial bone at the follow-up examination.

Mazzocco et al, [33] studied the bone volume changes after immediate implant placement with simultaneous grafting by anorganic bovine bone with or without flap elevation in non-molar region and reported a mean reduction of around 0.5 mm in height and width. Another aim of this study was to assess the effect of regenerative materials. We did not find significant differences in bone height changes between the two study groups. The effect of different materials in regenerative therapies combined with immediate implantation has been evaluated in several clinical studies. Although many authors advise the use of bone material with immediate implant placement, the evidence for this additional treatment is unclear from controlled clinical trials [17,18,34]. In recent animal studies, Araújo and Lindhe [18] and Araújo et al. [39] described reduction in dimensions of the post extraction site by use of deproteinized bovine bone mineral combined with collagen (Bio-Oss Collagen) [18,38]. Their results showed that usage of Bio-Oss® Collagen in immediate implantation decreased buccal vertical resorption from 1.3±0.7 mm to 0.1±0.5 mm. Clementini et al. [37] concluded that there is not enough evidence for any statement that immediate implant placement, in association with a regenerative technique, may be useful to prevent alveolar reduction. Although the differences between approaches were small, it seems that there is a trend towards better outcome with combined use of regenerative techniques. However, it is not clear that regenerative techniques are helpful for patients in long- term or not. Moreover, flapless approach used in our study did not prevent the occurrence of bone loss and its effect on bone remodeling was in concordance with the result of Novaes et al. [40]. They demonstrated that bone height dimensional changes were at least two times more pronounced when immediate implants were installed after elevation of mucoperiosteal flap. Nobuto et al, [41] studied microvascular responses after flap procedures in dogs. They found that resorption after elevation of the periosteum occurs due to insufficient blood circulation. However, flapless approach has some limitations including more exfoliation of the filing material, especially when the gaps are not covered with membrane or connective tissue, as in our study, so part of the material could be lost during the healing process.

## CONCLUSION

This study demonstrated that immediate implantation in fresh extraction socket together with xenograft failed to prevent bone resorption. The two different treatment modalities resulted in alterations of the vertical and horizontal dimensions of the buccal bone plate. More studies with larger sample sizes should be conducted for better elucidation of the impact of these two factors on buccal bone remodeling around dental implants.
